# The economic value of informal long-term care

**DOI:** 10.1093/eurpub/ckac130.121

**Published:** 2022-10-25

**Authors:** T Huis in ‘t Veld, D Korver, R Orhan, L Berenschot

**Affiliations:** Ecorys, Rotterdam, Netherlands

## Abstract

Informal care makes up a significant share of long-term care (LTC) and is likely to become even more important in the near future because of the ageing population, rising numbers of chronic patients and shortages of formal carers. At the same time, informal care is under pressure because of societal developments (shrinking family sizes, increased participation of women on the labour market, rising retirement age and geographical dispersion of families). These developments may further enhance the effect of informal care in terms of individuals’ quality of life and opportunity costs caused by work absence and productivity losses. Against this background, Ecorys has performed studies, to empirically estimate the social value of informal LTC and increase the awareness on its importance. The results of the study, commissioned by the European Commission, demonstrate that informal caregivers represent about 80% of all caregivers (measured in FTE). If paid for, these hours would cost between 2.4 and 2.7% of EU GDP. Currently, these costs are not paid and the actual costs of informal care are estimated at 1.05% of EU GDP, mostly in lost tax revenues. European countries vary greatly in the extent to which informal carers are supported by public policies. Only some countries have cash benefit schemes to compensate for the loss of income from employment. Specific benefits and leave entitlements to support informal caregivers are often insufficient or lacking. In several studies in the Netherlands, Ecorys found that total social costs of informal care are estimated at 2.54% of NL GDP (€22 billion/year) and informal caregivers perceive increasing economic and psychological burden and are at higher risk of depression and burn out. Government policies are developed to better support informal caregivers.

**Key messages:**

- Throughout the EU, most long-term care is provided by informal caregivers. Social costs of informal care represent 2.4 – 2.7% of EU GDP, actual financial costs are 1.05%.
- Informal caregivers suffer negative effects in terms of income and quality of life. Public policies to support informal caregivers vary widely throughout the EU.

